# An Introduction to Physical Measurements

**Published:** 1874-04

**Authors:** 


An Introduction to Physical Measurements, *with appendices on absolute Electrical Measurements, etc.* By Dr. F. Kohlrausch, Professor-in-Ordinary at the Grand Ducal Polytechnic School at Darmstadt, etc. Translated from the second German edition, by Thomas Hutchinson Waller, B.A., B.Sc., and Henry Richardson Proctor, F. C. S.—New York: D. Appleton & Co., 1874. Octavo; pp. 249. Cloth, Price not stated.

We confess to having been a little puzzled, on first glancing at the title page of this work, to comprehend exactly its drift and purpose, and lest some of our readers might find themselves in a similar quandary, we "rise to explain" by a few quotations from its table of contents, namely: Adjusting and testing a balance; determination of the sensativeness of a balance; density or specific gravity; calculation of the density of air, or of a gas, from its pressure and temperature; determination of heights by the barometer; freezing and boiling points of a thermometer; humidity of air; specific heat; modulus of elasticity; velocity of sound, by dust figures; spectrum analysis; focal length of a lens; saccharimetry by rotation of plane of polarization; moment of inertia; coefficient of torsion; magnetic inclination; Ohm's laws of galvanic currents; absolute system of electrical measurements; and numerous tables, *e.g.*, density of bodies—solids, fluids, gasses; percentage and specific gravity of various fluids, alcohol, acids, saline, solutions, etc., etc., and tables of squares, square-roots and reciprocals and logarithms.

